# Perceptions and Satisfaction of E-Health Applications: An Analytical Cross-Sectional Study in a Rural Health Center at Tiruvallur District, Tamil Nadu

**DOI:** 10.7759/cureus.66660

**Published:** 2024-08-11

**Authors:** Rex Vijay V, Praveen Kumar I, Buvnesh Kumar M, Sagetha J

**Affiliations:** 1 Department of Community Medicine, Saveetha Medical College and Hospital, Saveetha Institute of Medical and Technical Sciences (SIMATS), Saveetha University, Chennai, IND

**Keywords:** mobile health applications, telemedicine, digital health, healthcare delivery, rural health, user satisfaction, e-health

## Abstract

Introduction

E-health, defined as the utilization of information and communication technologies for health services, has become integral in enhancing healthcare delivery and accessibility. This study focuses on user satisfaction and perceptions of e-health applications in rural health centers, with special focus on Tamil Nadu, India. E-health technologies have proven to be effective in addressing challenges to healthcare accessibility and improving patient outcomes, at reduced costs. Despite these benefits, there is a need to understand user experiences in rural settings to optimize the implementation of e-health solutions.

Methods

A cross-sectional study was conducted among 383 patients registered in a non-communicable disease (NCD) clinic and specialty clinic in the rural health center of a tertiary care hospital in Tiruvallur district. Participants were selected using a consecutive sampling method from the NCD and specialty clinic registers. A semi-structured questionnaire was used to collect data on their perception and satisfaction with e-health applications. Data was entered in (Microsoft) MS Excel (Microsoft Corporation, Redmond, Washington, United States) and analyzed using IBM SPSS Statistics for Windows, Version 25 (Released 2017; IBM Corp., Armonk, New York, United States).

Results

The overall mean age was 49.45 ± 7 years. Among the study participants, females constituted 57.3% compared to males who constituted 42.7%. 58.3% of the participants had comorbid conditions. More than half of the study participants were educated up to the high school level. According to BG Prasad's classification, 86.9 % of the participants belonged to middle class and below. Among the study participants, more than half of them use their smartphones as devices for internet access to use e-health applications. The study participants who had no co-morbid conditions were 3.3 times the odds of having poor perception and satisfaction when compared to the other categories (OR = 3.3, CI = 2.1 - 5.1) in using e-health applications, and this difference was found to be statistically significant (p = 0.01).

Conclusion

This study's findings reveal that gender, socio-economic status, occupation, and the presence of comorbid illnesses play significant roles in shaping users' perceptions and satisfaction levels. This study's findings underscore the importance of tailored e-health interventions to address these barriers and enhance healthcare delivery in rural areas.

## Introduction

E-health applications, encompassing mobile health (mHealth) applications, telemedicine platforms, and electronic health records (EHRs), are revolutionizing healthcare delivery and access, designed to meet various healthcare needs. These applications provide tools for health monitoring, disease management, patient education, and communication between patients and providers by enabling remote healthcare services to overcome geographical barriers by reaching underserved populations. Patients can consult healthcare professionals without physical visits through telemedicine, enhancing accessibility and efficiency. Additionally, e-health applications facilitate the integration of healthcare services, real-time health data sharing, and better care coordination, ultimately improving patient outcomes [1,2]. 

E-health is defined as “the use of information and communication technologies (ICT) for health services and information”. This includes a wide range of services, such as electronic health records, telemedicine, mobile health applications, fitness applications, and online health education platforms. E-health aims to improve healthcare delivery by enhancing patient outcomes and increasing accessibility to health services, particularly in underserved areas [1].

E-health applications address significant challenges in the accessibility of health care, especially for rural and remote areas, by offering virtual consultations, remote monitoring, and continuous support. These applications empower patients to actively manage their health, ensuring timely medical attention and follow-up [3-5]. The quality of healthcare benefits greatly from e-health technologies through more accurate diagnoses, personalized treatment plans, and enhanced patient engagement. By providing comprehensive patient information and self-management tools, e-health applications lead to better health outcomes and higher patient satisfaction. They also increase the efficiency of the healthcare system by reducing the burden on facilities, decreasing wait times, and lowering costs [3,4].

Globally, e-health technologies have seen increased adoption in rural areas as a means to bridge the gap in healthcare accessibility [5]. For instance, In India, e-health initiatives have become crucial in delivering healthcare services to remote rural populations [2,6]. Tamil Nadu, one of the states in India, has been progressively adopting e-health technologies to improve healthcare accessibility in its rural areas, resulting in better health outcomes and patient satisfaction [5,6].

E-health significantly impacts the quality of healthcare received by the community by providing timely access to consultations, reducing the need for travel, and facilitating continuous monitoring [1]. In rural health centers, e-health applications have proven their effectiveness in managing chronic diseases, improving patient follow-up, and enhancing overall health service delivery [1]. This technology ensures that even the most remote population has access to quality healthcare, which is essential for improving health and quality of life [4]. The importance of e-health technologies in accessing healthcare in rural areas cannot be overstated, offering solutions to several barriers faced by rural populations, such as geographical isolation, shortage of healthcare professionals, and limited healthcare infrastructure [5]. By leveraging e-health, rural health centers can provide comprehensive healthcare services, ensuring timely and effective care, particularly in emergencies where immediate medical attention is required [1,6].

The benefits of using e-health in receiving quality healthcare are manifold. E-health applications facilitate real-time communication between patients and healthcare providers, enabling remote monitoring, and providing health information and education [2]. These technologies also help in reducing healthcare costs by minimizing the need for physical visits and hospitalizations [3]. Additionally, e-health empowers patients by giving them greater control over their health and medical data, leading to increased patient engagement and satisfaction [3].

Despite the recognized benefits of e-health, there is a need to understand user satisfaction and perception regarding its use in rural health centers. This study aims to fill this gap by exploring the experience of users in a rural health center in Tamil Nadu [2]. Understanding user satisfaction and perception is crucial for improving the design and implementation of e-health technologies, ensuring the needs of rural populations are met, and contributing to effective healthcare delivery [2]. The primary objectives of this study are to assess user perception and satisfaction with e-health applications in a rural health center in Tamil Nadu and to identify barriers to effective use of e-health in rural healthcare settings.

## Materials and methods

This was a cross-sectional analytical study undertaken in a tertiary care hospital in rural Tiruvallur, Chennai. The study was conducted over five months from July 2022 to December 2022. The study subjects comprised patients attending a non-communicable disease (NCD) clinic and specialty clinic using at least one e-health application and were willing to participate in the study by giving their valid consent. Those patients who did not give their valid consent were excluded from the study. Total participants included were 383 (N= 383).

Considering the prevalence of e-health applications usage from the study by Paradis et al. [7] as 47.4% with an alpha error of 0.05%, the power of study at 80% with a confidence interval of 95%, the minimum sample size for the study was calculated as 383 by using version 3.01 sample size calculator, which was rechecked using the formula: n = (Z_1 -α/2_)^2^ * P*Q/d^2^, where P = 47.4, making it n = 3.84*47.4*42.6/(5)^2^, and finally n = 383, wherein Z_1 -α/2_ is the standard normal variate (at 5% type 1 error (p < 0.05), making it 1.96; P is the expected prevalence from previous studies; Q = 1 - P; d is the allowable error; and n is the sample size.

Study participants were selected consecutively from NCD clinics held twice a week and specialty clinics held four days a week in the hospital, and the patients were interviewed at their respective clinics. Every alternative patient who visited the clinic was selected for the study; it was done until the minimum sample size was attained. If a patient was not willing to participate in the study, the next patient was included. Data collection was preceded by a training session for medical interns who conducted the interview.

A semi-structured survey questionnaire was developed to measure user perception and satisfaction among the e-health application users. The first part of the questionnaire was about system usability, functionality, perception of e-health application usage, and overall satisfaction. The second part was about the barriers and limitations of using the e-health applications. The questionnaire was prepared by referring to an in-depth literature review of previous studies on this topic of study and validated by two experts in this field.

The data collected using the interview method was entered in MS Excel (Microsoft Corporation, Redmond, Washington, United States) and analyzed using IBM SPSS Statistics for Windows, Version 25 (Released 2017; IBM Corp., Armonk, New York, United States). Descriptive statistics were used to analyze the baseline details, and a chi-square test was used to find the association between perception and satisfaction of using e-health applications and socio-demographic variables. Binary logistic regression was done to remove the confounders. The study was conducted after obtaining ethical approval from the Institutional Ethical Committee (SMC/IEC/04/001). Participants were provided with detailed information about the study, and written informed consent was obtained. Participants' identity was always kept confidential, and the data was used only for research purposes.

## Results

Table 1 shows the distribution of socio-demographic details of the study participants. The overall mean age was 49.45 ± 7 years. Among the study participants, females constitute 57.3% compared to males who were 42.7%. 58.3% of the participants had at least one comorbid condition. More than half of the study participants were educated up to the high school level. According to Brahm Govind (BG) Prasad's classification for October 2023 [8], 86.9 % of the participants belonged to middle class and below. More than half of the participants use their smartphones to access the Internet and e-health applications in their day-to-day lives. About more than half of the study participants were employed as daily wagers.

**Table 1:** Socio-demographic distribution of study participants (N=383) *Socio-economic status according to the modified Brahm Govind (BG) Prasad scale, October 2023 **Upper class and upper middle were categorized as upper class. ***Middle class, lower middle class, and lower class were categorized as lower class.

Sl. No	Variable	Categories	Frequency N (%) n = 383
1	Gender	Male	164 (42.7)
		Female	219 (57.3)
2	Educational status	Up to high school	235 (61.3)
		High school and above	148 (38.7)
3	Socio-economic status*	Upper class^**^	50 (13.1)
		Middle class and below^***^	333 (86.9)
4	Occupation	Employed for daily wages	228 (59.5)
		Professional	155 (40.5)
5	Devices used for Internet access	Smartphone	217 (56.6)
		Others (multiple device use)	166 (43.4)
6	Comorbid status	Yes	223 (58.3)
		No	160 (41.7)

Table 2 shows the perceptions of using e-health applications among the study participants. More than half of the participants (67.5%) found e-health applications difficult to use. Among the study participants, 31.5% reported that the amount of time spent on using e-health applications was acceptable, 44.8% viewed e-health applications as an acceptable way to receive healthcare services, and 50.26% expressed satisfaction with the quality of service provided via the applications. More than half of the study participants (56.25%) felt comfortable communicating with their healthcare providers via e-health applications. Among the study participants, 53.12% found it easy to track their lifestyle habits. and 48.95% reported being satisfied with the overall quality of service provided via the application.

**Table 2:** Perception and satisfaction of using e-health applications among study participants (N=383)

Sl No	Variable	Perceptions of using e-health applications n = 383 (%)	
		Yes	No
1	It was easy to use	125 (32.5)	258 (67.5)
2	Acceptability amount of time spent on using e-health apps	120 (31.51)	263 (68.49)
3	E-health apps are an acceptable way to receive healthcare services	172 (44.8)	211 (55.2)
4	Satisfaction with the quality of service being provided via the app	193 (50.26)	190 (49.74)
5	Feeling comfortable communicating with my healthcare provider via E – health apps	216 (56.25)	167 (43.75)
6	It was easy to track lifestyle habits through the app	204 (53.12)	179 (46.88)
7	Overall, I am satisfied with the quality of service being provided via the app	187 (48.95)	196 (51.05)

**Figure 1:**
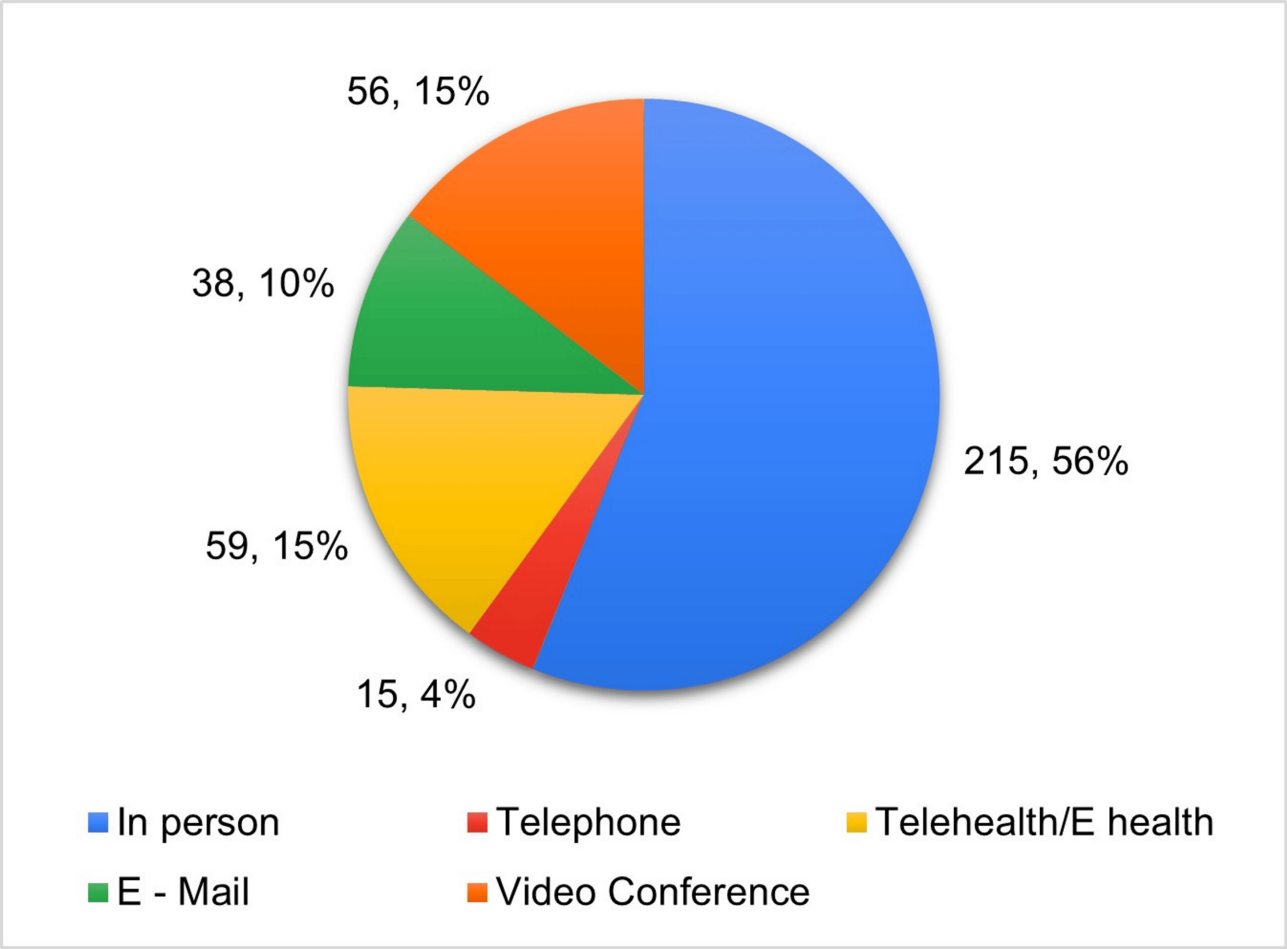
Way of communication with doctors /healthcare providers (N=383)

Figure 1 shows the ways of communication with doctors/healthcare providers; among the study participants, about 215 communicated with the healthcare providers in person and 38 respondents communicated with the healthcare providers through e-mail.

**Figure 2:**
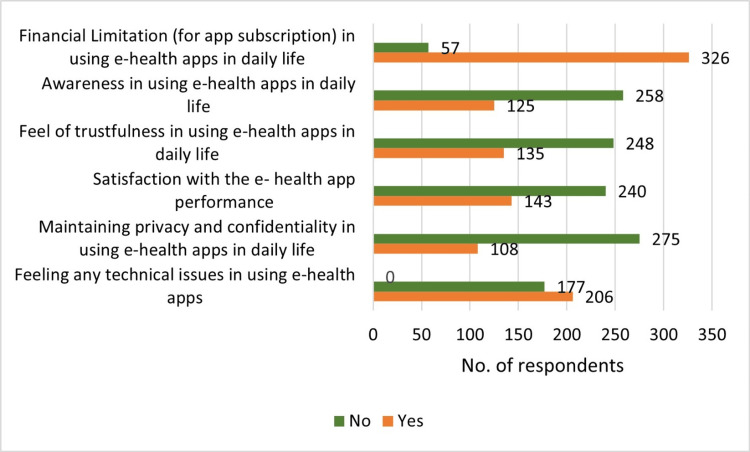
Barries of using e-health applications (N=383)

Figure 2 shows the main barriers identified in the study regarding the use of e-health applications. Out of 383 study participants, 297 reported experiencing technical issues while using e-health applications. Among the study participants, 108 expressed concerns about maintaining privacy and confidentiality while using e-health applications, highlighting the importance of data security and privacy protection in digital healthcare. Among the study participants, about 143 reported dissatisfaction with the performance of e-health applications, pointing to issues regarding application functionality, user experience, or service delivery. One hundred and thirty-five participants mentioned a lack of trustfulness in using e-health applications, indicating concerns about the reliability, accuracy, or credibility of the information and services provided through the applications. One hundred and twenty-five participants reported a lack of awareness in using e-health applications in daily life in utilization of digital health technologies. Three hundred and twenty-seven participants had financial limitations, such as subscription costs as a barrier to using e-health applications for digital healthcare services.

Table 3 shows the association of socio-demographic variables with perception and satisfaction while receiving health care delivery through e-health applications. Among the gender categories, 50.7% of females had a poor perception of receiving healthcare through e-health applications compared to males, and this difference was not found to be statistically significant. Among the socio-economic status categories, 48.6% of study participants belonged to the middle class and below had poor perceptions about receiving healthcare through e-health applications compared to other categories, and this difference was found to be statistically significant. Among the study participants, 50.9% who were employed as daily wagers had a poor perception of receiving health care through e-health applications compared to the other category and this difference was statistically significant. Among the study participants, 62.5% who did not have any comorbid conditions had a poor perception of receiving healthcare through e-health applications compared to those who had comorbid conditions, and this difference was not found to be statistically significant.

**Table 3:** Association of socio-demographic variables with perception and satisfaction in receiving health care through e-health applications (N=383) *p-value <0.05 is significant, Chi-square test

Sl. No	Variable	Category	Perception and satisfaction in receiving healthcare through e-health applications		Unadjusted OR (95% CI)	p-value
			Good n = 206 (%)	Poor n = 177 (%)		
1	Gender	Male	98 (59.8)	66 (40.2)	1.526 (1.013 – 2.298)	^*^0.04
		Female	108 (49.3)	111 (50.7)		
2	Education	Up to high school	114 (48.5)	121 (51.5)	0.573 (0.377 – 0.872)	0.09
		High school	92 (62.2)	56 (37.8)		
3	Socio-economic status	Upper class^*^	35 (70)	15 (30)	2.211 (1.163 – 4.201)	*0.01
		Middle class and below^*^	171 (51.4)	162 (48.6)		
4	Occupation	Employed for daily wages	112 (49.1)	116 (50.9)	0.627 (0.414 – 0.948)	^*^0.02
		Professional	94 (60.6)	61 (39.4)		
5	Devices used for Internet access	Smartphone	110 (50.7)	107 (49.3)	0.751 (0.499 – 1.126)	0.16
		Others (use of multiple devices)	96 (57.8)	70 (42.2)		
6	Comorbid status	Yes	146 (56.5)	77 (43.5)	3.161 (2.071 – 4.822)	^*^0.01
		No	60 (37.5)	100 (62.5)		

Table 4 shows a binary logistic regression of factors affecting perception and satisfaction about using e-health applications. After adjusting for gender, education, socio-economic status, occupation, devices used for internet access, and co-morbid status binary logistic regression was done. The study participants who were educated up to high school were 1.6 times the odds of having poor perception and satisfaction when compared to the other category (OR = 1.6, CI = 1.1 - 2.5) in using e-health applications, and this difference was found to be statistically significant. Among the study participants who did not have co-morbid conditions were 3.3 times odds of having poor perception and satisfaction when compared to the other category (OR = 3.3, CI = 2.1 - 5.1) in using e-health applications, and this difference was found to be statistically significant. Other independent factors were not statistically significant.

**Table 4:** Binary logistic regression of factors affecting perception and satisfaction in using e-health applications (N=383) *p-value <0.05 is significant, binary logistic regression ^1^Reference category

Sl. No	Variable	Category	Adjusted OR (95% CI)	p-value
1	Gender	Male	0.659 (0.434 – 1.002)	0.05
		Female^ 1^		
2	Education	Up to high school	1.651 (1.073 – 2.541)	^*^0.02
		High school^1^		
3	Socio-economic status	Upper class	0.642 (0.303 – 1.359)	0.24
		Middle class and below^ 1^		
4	Occupation	Employed for daily wages	1.319 (0.832 – 2.090)	0.23
		Professional^1^		
5	Devices used for Internet access	Smartphone	1.047 (0.669 – 1.638)	0.84
		Others (use of multiple devices)^1^		
6	Comorbid status	No	3.328 (2.147 – 5.157)	*0.01
		Yes^1^		

## Discussion

Socio-demographic distribution of the study participants

The socio-demographic profile of our study participants reveals an average age of 49.45 ± 7 years, with females constituting 57.3% and males 42.7%. According to the Census 2011, the percentage of females in the Tiruvallur district comes to around 49.8% and males 50.1% [9]. In comparison, our study showed a slightly higher proportion of females than males [9].

Perception of using e-health applications among study participants

In our study, 67.5% of participants found e-health applications difficult to use. This is higher compared to findings by Melin et al., where the majority of users reported satisfactory ease of use for mobile health applications. The higher difficulty reported in our study may be attributed to differences in the user interface or the specific design and functionality of the applications studied [10]. About 31.5% of participants in our study reported that the amount of time spent on e-health applications was acceptable. Lahariya et al. found that the majority of users were satisfied with the time efficiency of health services at Mohalla Clinics, indicating a need for e-health apps to improve their efficiency to match traditional health service experiences [11].

In our study, 44.8% of participants viewed e-health applications as an acceptable way to receive healthcare services. Albaghdadi and Al Daajani reported higher acceptability levels in their study on telemedicine in Jeddah, suggesting that regional differences and cultural factors may influence the acceptance of e-health technologies [4]. Among our participants, 50.26% expressed satisfaction with the quality of service provided through the application. Alharbi and Almufarij found similar satisfaction levels during the COVID-19 pandemic in Saudi Arabia, indicating a general positive trend in the perception of e-health services during crises [12].

In our study, 56.25% of participants felt comfortable communicating with healthcare providers through e-health applications. This aligns with findings by Paradis et al., where a significant number of users reported comfort in using smartphone health applications for communication with healthcare providers [7]. Our study found that 53.12% of participants found it easy to track their lifestyle habits through the application. This is comparable to the findings by Chan and Honey, who reported good usability and acceptability of mobile digital apps for mental health, indicating that e-health applications are effective tools for monitoring personal health behaviors [13]. In our study, 48.95% of participants reported being satisfied with the overall quality of service provided through the application. This is in line with the findings from Duplaga and Turosz, where user satisfaction with e-health applications was significant during the early phase of the COVID-19 pandemic in Poland [2].

A study conducted by Polinski et al. (2016) found that patients generally expressed satisfaction with telehealth visits, appreciating the convenience and time-saving aspects (Polinski et al., 2016). Similarly, Mann et al. (2020) highlighted that telemedicine has transformed healthcare delivery, especially during the COVID-19 pandemic, enhancing patient engagement and satisfaction [14,15]. These studies indicate a positive perception and moderate level of satisfaction with e-health applications among e-health application users.

Barriers to use e-health applications

A significant barrier was identified with the concern over privacy and data security. About 108 participants expressed concerns about maintaining privacy and confidentiality. This aligns with the findings by Garg and Brewer (2011), who identified security and privacy as major issues in telemedicine. Additionally, Alenoghena et al. (2023) noted the importance of robust data protection mechanisms to alleviate these concerns [16,17]. In our study, privacy issues were also adequately addressed; in contrast to several international studies that indicate patients are concerned about privacy, the majority of participants disagreed that telemedicine may result in privacy breaches [4,18]. There could be cultural reasons for this discrepancy. Our study found that 135 participants lacked trust in e-health applications, possibly due to concerns about the reliability and accuracy of information. Similar results were noted by Kao and Liebovitz (2017), who discussed the variability in the quality and trustworthiness of mobile health applications [19].

Financial limitations, such as subscription costs, were cited by 327 participants as a barrier. This concern is echoed by Singh et al. (2016), who highlighted the cost implications of telehealth for high-need, high-cost populations [20]. Technical issues were reported by 207 participants, indicating challenges with app functionality and user experience. This is consistent with the findings of Dietrich et al. (2018), who identified technological limitations as a barrier to effective telemedicine adoption [21].

Association of socio-demographic variables with perception and satisfaction in receiving health care through e-health applications

This study explores the association of various socio-demographic factors with the perception and satisfaction of receiving healthcare through e-health applications. The findings reveal that gender, socio-economic status, occupation, and the presence of comorbid illnesses play significant roles in shaping users' perceptions and satisfaction levels.

Our study found that 50.7% of females had a poor perception of receiving healthcare through e-health applications compared to males. However, this difference was not statistically significant. This contrasts with findings by Alharbi et al., who noted that gender differences significantly influenced perceptions and satisfaction with mobile health applications during the COVID-19 pandemic in Saudi Arabia [12]. A significant finding in our study was that 48.6% of participants from the middle class and below had poor perception of e-health applications, which showed a statistically significant difference. This aligns with Lahariya et al., who reported that lower socio-economic groups in India faced more challenges in accessing and utilizing health services, which could influence their perception of e-health applications [11]. Participants employed as daily wagers showed a higher incidence (50.9%) of poor perception towards e-health applications, which was found statistically significant. This finding is consistent with Bassi et al., who found that occupation and employment status significantly influenced the usage and satisfaction levels of mobile health interventions in India [22].

Our study indicated that 62.5% of participants without comorbid conditions had poor perception of e-health applications, although this was not statistically significant. This is somewhat divergent from the findings of Melin et al., where individuals with chronic conditions often had better satisfaction and perception of mobile health applications due to their regular need for continuous monitoring and support [10].

The logistic regression analysis revealed that participants with education up to high school had 1.6 times higher odds of having a poor perception and satisfaction compared to those with higher education, a statistically significant difference. This finding is corroborated by research from Duplaga and Turosz, who found that lower education levels were associated with poorer perception and satisfaction with e-health services [2]. Participants without comorbid conditions had 3.3 times higher odds of poor perception and satisfaction, which was statistically significant. This is in line with the findings by Chan and Honey, who noted that individuals with no comorbid conditions might not perceive the need for regular use of e-health applications as strongly as those with ongoing health issues [13].

E-health applications provide an array of advantages and drawbacks. Indeed, by offering prompt and practical services, these applications can greatly improve access to healthcare, especially for people living in rural or underserved areas. Studies showing enhanced patient satisfaction and better telemedicine access added legitimacy to this [23]. A further advantage mentioned in previous studies was the time reduction from cutting down travel and waiting periods at the hospital [4,24]. In contrast, telemedicine presents some difficulties. If the digital gap is not carefully addressed, it may worsen health inequities, especially for elderly people and those living in rural or low-income areas [4,25].

This study’s strengths include its large sample size, which provides robust statistical power for the findings, and the training provided to medical interns conducting interviews, enhancing the quality and consistency of data collection. However, several limitations affect the study's generalizability and accuracy. The reliability of self-reported data may introduce subjective bias, and the limited duration of five months might not capture long-term trends in e-health application usage. Additionally, the variability in application design and functionality was not controlled, potentially influencing user perceptions and satisfaction.

Limitations of the study

To address these limitations, future studies should expand their geographic scope to include both rural and urban locations, enhancing generalizability. Longitudinal studies are recommended to understand better the long-term impact of socio-demographic variables on e-health applications perception and satisfaction. Research should also standardize application features to isolate the impact of design and functionality on user experience. Targeted educational and support interventions are needed to improve usability among less educated and economically disadvantaged groups, and technological training should be provided to both healthcare providers and patients to enhance technological literacy and ease of use of e-health applications.

## Conclusions

Our study highlights the critical influence of socio-demographic variables on the perception and satisfaction of e-health apps. While the findings highlight that gender, socio-economic status, co-morbid status, and education level are key determinants, it is evident that targeted interventions are needed to enhance the perception, acceptability, and usability of e-health technologies, especially among vulnerable and less-privileged groups. Addressing these factors can help bridge the digital divide and ensure more equitable access to healthcare services, ultimately improving health outcomes for all population segments.

## Appendices

E-health questionnaire

Socio-Demographic Variables

**Table 5:** Socio-demographic details

Sl No	Variable	Responses
1	Name of the person	
2	Age	
3	Gender	Male/Female
4	Educational status	Up to high school degree/high school degree and above
5	Occupation	Employed for daily wages/Professional
6	Devices used for Internet access	Smartphone/others (use of multiple devices)
7	Socio-economic status	Class 1/2/3/4/5/6
8	Comorbid status	Yes/No

Perceptions of Using E-Health Applications

**Table 6:** Perception of using e-health apps

Sl No	Perception of using e-health apps	Responses
1	It was easy to use	Yes/No
2	The time spent using it has been acceptable	Yes/No
3	I find E- health apps an acceptable way to receive healthcare services	Yes/No
4	Satisfaction with the quality of service being provided with the app	Yes/No
5	I feel comfortable communicating with my healthcare provider via E - health apps	Yes/No
6	Overall, I am satisfied with the quality of service being provided via the app	Yes/No
7	It was easy to track lifestyle habits through the app	Yes/No
8	How do you communicate with your doctors/health care Providers usually	Telephone/In person/Video – conference/Email/Telehealth(eHealth)

Barriers of Using E-Health Apps

**Table 7:** Barriers of using e-health apps

Sl No	Barriers of using e-health apps	Responses
1	Do you feel any technical issues in using e-health apps?	Yes/No
2	Is your privacy and confidentiality maintained in using e-health apps in daily life?	Yes/No
3	Are you satisfied with the e-health app's performance?	Yes/No
4	Do you feel trustful in using e-health apps in daily life?	Yes/No
5	Are you aware of using e-health apps in daily life?	Yes/No
6	Do you find any financial Limitations (for app subscription) in using e-health apps in daily life?	Yes/No
